# Determination of Total Antioxidant Status and Some Physicochemical Properties of Organic and Conventional Floral Honeys

**DOI:** 10.3390/molecules31162862

**Published:** 2026-08-16

**Authors:** Esra Demirata, Burak Demirhan, Buket Er Demirhan

**Affiliations:** 1 Division of Food Analysis and Nutrition, Graduate School of Health Sciences, Gazi University, 06330 Ankara, Türkiye; esra.demirata@gazi.edu.tr; 2 Department of Pharmaceutical Basic Sciences, Faculty of Pharmacy, Gazi University, 06330 Ankara, Türkiye; bdemirhan@gazi.edu.tr

**Keywords:** honey, organic, antioxidant, functional food

## Abstract

Honey is a natural food widely consumed due to its nutritional and functional properties. With the growing consumer interest in organic foods, evaluating the composition and quality parameters of organic and conventional honey products available on the market has become increasingly important. For this purpose, a total of 92 organic and conventional floral honey samples obtained from four different firms in Türkiye were analyzed for total antioxidant status (TAS), total oxidant status (TOS), total phenolic content, proline content, diastase activity, hydroxymethylfurfural (HMF) level, pH, electrical conductivity, color, °Brix, and moisture content. In the floral honey samples, mean values of TAS, TOS, and total phenolic content ranged from 0.48 to 0.93 mmol Trolox equivalent/L, 12.03 to 56.76 µmol H_2_O_2_ equivalent/L, and 97.17 to 153.00 mg GAE/kg, respectively. Furthermore, the mean values of proline content, diastase number, HMF content, electrical conductivity, and moisture content ranged from 314.27 to 699.81 mg/kg, 8.57 to 28.50, 3.24 to 23.30 mg/kg, 0.20 to 0.28 mS/cm, and 15.70 to 17.39%, respectively, all of which were within the limits specified in the Turkish Food Codex. Additionally, the mean pH and °Brix values ranged from 3.85 to 4.23 and 80.81 to 82.43%, respectively, while the color ranged from white to light amber.

## 1. Introduction

Honey has been regarded as an important natural food throughout history due to its health-promoting effects, therapeutic properties, and biological activities [1]. As a complex natural product, it contains primarily carbohydrates [2]. A wide range of bioactive components are present in honey, including pigments such as carotenoids and anthocyanins, various polyphenolic compounds such as phenolic acids and flavonoids, amino acids, vitamins C and E, enzymes, organic acids, and minerals [3]. Variations in the composition of these compounds cause differences in the color, taste, viscosity, and therapeutic properties of honey [4]. Evaluating the chemical composition and physicochemical properties of honey is considered an important requirement for establishing quality standards. Analysis of honey for moisture, color, pH, total soluble solids, acidity, ash content, electrical conductivity, HMF, and mineral elements provides important data for evaluating honey quality and ensuring it meets regulatory standards [5]. Due to these biological properties, honey is considered a functional food [3]. In addition to its widespread use as a food sweetener, honey is well known for its antibacterial and antifungal effects [6].

Honey’s antioxidant activity refers to its capacity to inhibit oxidative reactions occurring in food systems. Oxidative reactions cause quality losses and undesirable changes in food and are also associated with the development of various risks in human health, such as chronic diseases and cancer. Honey’s antioxidant capacity stems from its natural bioactive components, primarily flavonoids and phenolic acids, as well as polyphenolic compounds, enzymes such as glucose oxidase and catalase, ascorbic acid, carotenoid-like compounds, organic acids, proteins, and amino acids [7]. Commonly present in plants as well as plant-derived foods and beverages, phenolic compounds are defined as secondary metabolites. These compounds exhibit a wide chemical diversity, ranging from simple structures like gallic acid and caffeic acid to more complex polyphenolic structures like flavonoids [8,9]. In human nutrition, phenolic acids, flavonoids, and tannins are considered the most important classes of phenolic compounds [10]. Indeed, it is reported that the therapeutic potential effects of honey are largely related to its antioxidant capacity against reactive oxygen species (ROS) [11,12].

The botanical origin, geographical locations, and environmental quality (soil, water, air), along with recent climatic variations, are well known to drive year-to-year fluctuations in honey quality [13]. Honey quality is fundamentally characterized by its purity, which can be verified through key physicochemical parameters, namely moisture content, pH, free acidity, total soluble solids, sugar profile, color intensity, 5-hydroxymethylfurfural, and amino acid content. Consequently, evaluating these properties is essential for establishing both authenticity and quality, factors that directly determine market value and consumer acceptance [14].

Oxidative stress is a condition resulting from an imbalance between the formation of ROS and the antioxidant system’s ability to neutralize these compounds. The pathology of a number of diseases, including metabolic, vascular, and neural disorders, can be significantly influenced by oxidative stress [15]. Therefore, honey stands out as a natural food with strong antioxidant properties thanks to its phenolic compounds and flavonoids [16].

Organic honey production is an ecologically based production system that aims to protect the balance of agricultural ecosystems, support biodiversity, ensure the sustainable use of natural resources, improve environmental quality, and protect animal welfare and human health [17]. Limited data suggest that organic food products may have higher nutritional value compared to conventional products [18]. Therefore, consumers tend to prefer organic products because they associate them with a healthier and more sustainable lifestyle [19].

There are very limited studies comparing organic and conventional floral honeys in terms of phenolic compounds, antioxidant/oxidant status, and physicochemical quality parameters in Türkiye. Therefore, this study aimed to compare the biological and physicochemical properties of organic and conventional floral honeys by determining total antioxidant status (TAS), total oxidant status (TOS), total phenolic content, proline content, diastase activity, pH, color, HMF content, electrical conductivity, moisture content, and °Brix. To the best of our knowledge, the present study provides the first comprehensive dataset for organic and conventional honeys of Turkish origin, offers valuable foundational data to the existing literature, and can serve as a useful reference for future research and consumers.

## 2. Results and Discussion

### 2.1. TAS and TOS Values of Honey Samples

A total of 92 honey samples comprising organic (A_1_, B_1_, C_1_, D_1_) and conventional (A_2_, B_2_, C_2_, D_2_) variants sourced from four different firms in Türkiye were subjected to TAS and TOS analyses (Table 1). It was determined that the mean TAS values of organic honey samples of firms B_1_ and C_1_ were higher compared to those of conventional samples. The mean TAS values of organic honey samples ranged between 0.70 and 0.93 mmol Trolox equivalent/L, whereas the mean values of conventional honey samples ranged between 0.48 and 0.89 mmol Trolox equivalent/L. The highest mean TAS value was detected in organic firm B_1_, while the lowest mean TAS value was found in conventional firm C_2_. Statistically significant variations in TAS values were observed between samples B_1_ and B_2_, as well as C_1_ and C_2_ (*p* < 0.05).

Honey is considered a natural dietary antioxidant [11]. Since plant-based foods contain numerous and diverse antioxidant compounds, and interactions among these compounds may exert synergistic or antagonistic effects on total antioxidant capacity, determining the TAS value, which reflects the total capacity of foods to neutralize free radicals, is important [20]. The 2,2′-azino-bis-3-ethylbenzothiazoline-6-sulfonic acid (ABTS) and 2,2-diphenyl-1-picrylhydrazyl (DPPH) are effective methods for evaluating the overall antioxidant potential of honey, reflecting the general activity of its antioxidant components, including polyphenols, flavonoids, and phenolic acids. It is noted that while DPPH targets a single free radical, ABTS covers a wider range [21]. The ABTS method is reported to be good at testing the antioxidant activity of foods containing hydrophilic and highly pigmented compounds, and can screen lipophilic and hydrophilic substances [22,23]. Studies reported in the literature demonstrate that honey possesses significant antioxidant activity and that this activity may vary depending on botanical origin, geographical source, and production system. Herken et al. [20] determined the mean TAS values of certified and uncertified Turkish honeys as 0.216 and 0.087 mmol Trolox equivalent/L, respectively, and suggested that this difference may be related to the botanical origin of honey and its antioxidant compound content. Bouddine et al. [24] analyzed 23 organic honey samples obtained from different ecogeographical regions and found that total antioxidant activity values ranged between 34.18 and 131.20 mg AAE/g, with antioxidant capacity varying according to plant sources. In a study conducted by Castro et al. [25], 43 organic and 24 conventional honey samples collected from the Sierra Nevada de Santa Marta region of Colombia were analyzed for antioxidant capacity using the Trolox Equivalent Antioxidant Capacity (TEAC) and Ferric Reducing Antioxidant Potential (FRAP) methods. The antioxidant capacity values determined by the FRAP assay ranged from 0.48 (Conventional) to 0.51 mmol Trolox/kg honey (Organic), whereas the mean values obtained by the TEAC assay ranged from 0.66 mmol Trolox/kg honey (Conventional) to 0.75 mmol Trolox/kg honey (Organic).

In this study, the mean TOS values of organic honey samples were found to range between 12.03 and 56.76 µmol H_2_O_2_ equivalent/L, whereas those of conventional honey samples ranged between 14.26 and 53.43 µmol H_2_O_2_ equivalent/L. The highest mean TOS value was detected in organic firm D_1_, while the lowest mean TOS value was observed in organic firm A_1_. In our study, the mean TOS values of honey samples of A_2_, B_2_, and C_2_ were found to be significantly higher (*p* < 0.05) compared to A_1_, B_1_, and C_1_. In a study conducted by Herken et al. [20], the TOS values of certified and uncertified Turkish honey samples were investigated. The researchers analyzed 17 certified honey samples and eight uncertified honey samples obtained from different regions of Türkiye. The mean TOS values of the certified and uncertified samples were determined to be 8.585 and 1.144 μmol H_2_O_2_ equivalent/L, respectively. In another study, Akyol et al. [26] collected honey samples from beekeepers in Türkiye and reported that the TOS values of honey samples originating from various provinces of Türkiye ranged from 3.922 ± 0.163 to 13.613 ± 0.339 μmol H_2_O_2_ equivalent/L.

### 2.2. Total Phenolic Content Values of Honey Samples

According to our findings, the mean total phenolic content of organic and conventional honey samples ranged from 97.17 to 124.14 mg GAE/kg and 98.60 to 153.00 mg GAE/kg, respectively (Table 1). The mean total phenolic contents of A_2_, B_2_, and D_2_ samples were found to be statistically significantly higher (*p* < 0.05) than those of A_1_, B_1_, and D_1_ samples. The highest mean total phenolic content was found in conventional honey from firm B2, while the lowest mean total phenolic content was found in organic honey from firm C_1_.

Phenolic compounds are aromatic phytochemicals with significant antioxidant activity in honey. They are natural products of secondary plant metabolism and reach honey through honeybees. Their range in honey is also very wide [27]. The national literature contains various studies investigating total phenolic contents. Ekici et al. [28] determined the total phenolic content of 20 pine honey samples collected from the Muğla and Marmaris regions to range between 35.30 and 92.87 mg GAE/100 g. In a study conducted by Herken et al. [20], the mean total phenolic contents of certified and uncertified Turkish honeys were determined as 17.144 and 10.930 mmol gallic acid equivalent/L, respectively. Akgün et al. [29] reported the overall mean phenolic content of multifloral, chestnut, acacia, and rhododendron honeys as 0.15 ± 0.10 mg GAE/g. Kara et al. [30] reported that the total phenolic content of honey samples from the Kırklareli region ranged from 54 to 88 mg GAE/100 g. Kara et al. [31] evaluated 24 floral honey samples from the Tokat region and found that their total phenolic content ranged between 254.14 and 776.94 μg GAE/g. In addition, Karlıdağ [32] reported that the mean total phenolic content of multifloral honeys from Malatya province ranged from 19.47 to 29.33 mg GAE/100 g. Ucuncu et al. [33] stated that the total phenolic content of 15 flower honey samples obtained from Gümüşhane province ranged from 9.21 to 98.25 mg GAE/100 g.

Similarly, several studies have been conducted at the international level regarding the total phenolic content. Stanojević et al. [27] reported that the total phenolic content of the analyzed honey samples ranged from 76.5 to 145.9 μg GAE/g. The highest total phenolic content (145.9 μg GAE/g) was found in conventional meadow honey, whereas the lowest content (76.5 μg GAE/g) was observed in conventional acacia honey. The authors found that conventional honey samples from different botanical origins generally contained higher levels of total phenolics than their organic counterparts, with the exception of acacia honey. Halagarda et al. [34] determined the mean total phenolic contents of organic and conventional honey samples of Polish origin to be 6.79 and 7.37 mg GAE/100 g, respectively. In a study investigating honey samples obtained from organic and conventional production systems in Colombia, the total phenolic content was reported as 410.51 mg GAE/kg in organic and 416.53 mg GAE/kg in conventional honeys; however, this difference was not statistically significant [25]. Moise et al. [35] reported a mean phenolic content of 54.22 ± 0.43 mg GAE/100 g in heather honey samples originating from Romania. In a study on Brazilian honey samples, Do Nascimento et al. [36] reported total phenolic contents ranging from 26.0 to 100.0 mg GAE/100 g. Furthermore, Gašić et al. [37] determined total phenolic contents of 58 polyfloral honeys in Serbia to range between 0.03 and 1.39 mg GAE/g.

### 2.3. Physicochemical Parameters of Honey Samples

#### 2.3.1. Proline Content

**Figure 1:**
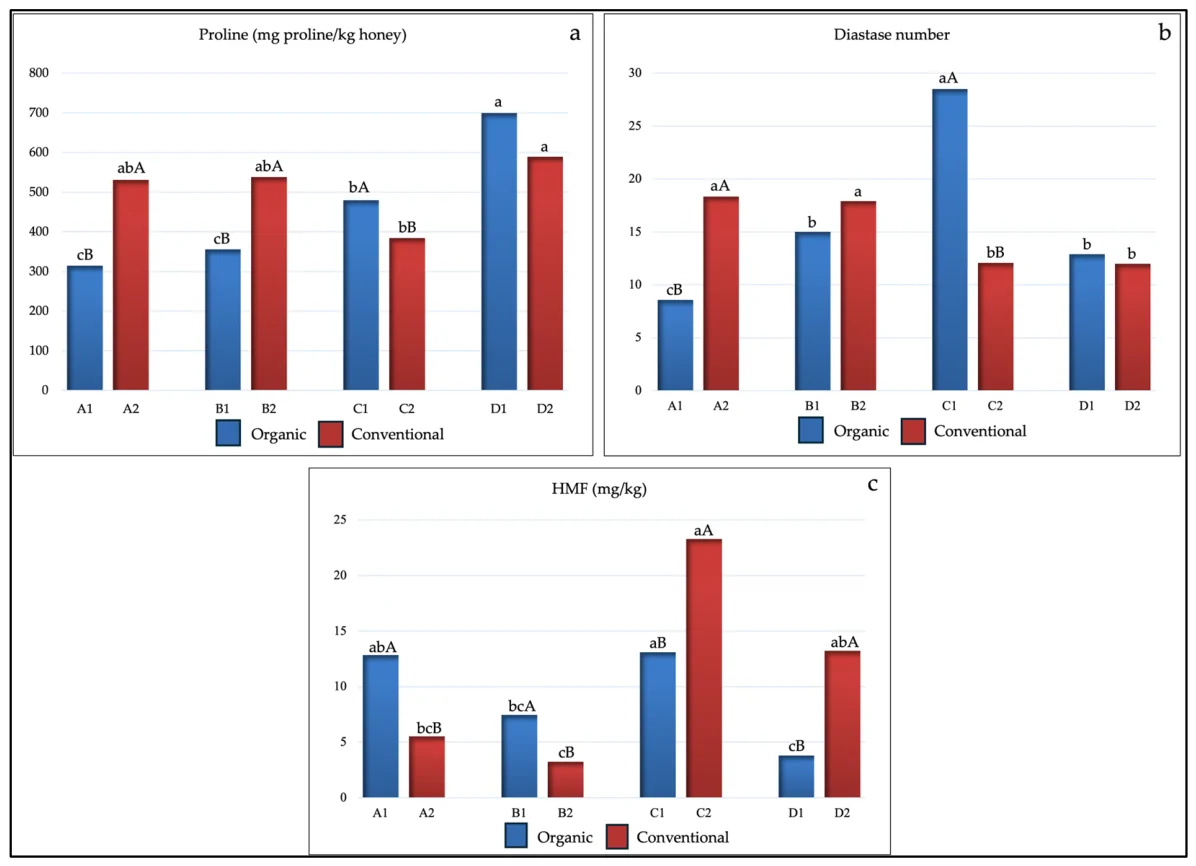
Proline content (**a**), diastase number (**b**), and HMF values (**c**) of organic and conventional honey samples. Different lowercase letters (a–c) above the bars indicate statistically significant differences among firms within the same production group (organic or conventional) (*p* < 0.05). Different uppercase letters (A and B) indicate statistically significant differences between the organic and conventional samples of the same firm (*p* < 0.05); the absence of an uppercase letter indicates that the difference between organic and conventional samples for that specific firm is not statistically significant (*p* > 0.05).

Proline is an indicator of the total amino acid content present in honey. It is considered one of the quality parameters of honey and can be used as a criterion for determining honey maturity and sugar adulteration [38]. According to the TFC, the proline content of floral honeys must be at least 300 mg/kg [39]. In the present study, the mean proline values of organic and conventional honey samples ranged between 314.27 and 699.81 mg/kg and 384.05–589.16 mg/kg, respectively (Table 2, Figure 1). The highest mean proline value was detected in organic firm D_1_, while the lowest value was observed in organic firm A_1_. In both organic and conventional honey samples, mean proline levels were consistent with the limit specified in the TFC.

A review of studies conducted in Türkiye reveals that the proline content of honey samples varies over a wide range. Bideci and Karasalihoğlu [40] reported that proline concentrations in honey samples collected from 40 producers in Kastamonu province ranged from 9.20 to 1176.10 mg/kg, with a mean value of 464.69 ± 218.14 mg/kg. Similarly, Yetkin et al. [41] determined proline contents ranging from 568 to 758 mg/kg in eight multifloral and monofloral honey samples collected from different regions of Rize. Ucuncu et al. [33] reported that the proline content of 15 flower honey samples obtained from Gümüşhane province varied between 562.66 and 1466.15 mg/kg. Özkan and Dodoloğlu [42] analyzed 21 honey samples from Erzurum province and found proline values ranging from 417 to 1449 mg/kg, with a mean of 974.19 mg/kg.

In a study conducted in Malaysia, Hassan et al. [43] analyzed 36 honey samples and found that their proline contents ranged from 38.8 to 797.2 mg/kg. Gela et al. [44] determined the mean proline value of 214 ± 15 mg/kg in honey samples from Ethiopia. Findings from international studies indicate that the proline content of honey exhibits substantial variation.

#### 2.3.2. Diastase Number

Beyond enriching the therapeutic and nutritional function of honey, diastase (α- and β-amylase) constitutes a fundamental marker used to evaluate overall honey quality. It is also affected by the decrease in enzymatic activity due to temperature [45]. The diastase number in honeys, excluding baking honey, must be at least 8 according to the TFC [39]. In the present study, the mean diastase numbers of the organic and conventional honey samples were found to range between 8.57 and 28.50 and 12.00–18.33, respectively (Table 2, Figure 1). The highest mean diastase number was observed in organic honey firm C_1_, while the lowest mean diastase value was found in organic honey firm A_1_. All honey samples complied with the diastase number limits specified by the TFC, indicating a favorable quality status for honey.

In studies from Türkiye, Yetkin et al. [41] found that the diastase activity of eight honey samples collected from the Rize province varied between 9.01 and 53.95. Ucuncu et al. [33] reported diastase numbers ranging from 23.59 to 60.00, with a mean value of 54.15 ± 6.75, in 15 flower honey samples obtained from Gümüşhane province. Bideci and Karasalihoğlu [40] reported that diastase activity in honey samples in Kastamonu province ranged from 0.03 to 85.40, with a mean value of 14.4 ± 12.67. Studies conducted in Türkiye demonstrate that diastase activity can vary considerably among honey samples produced in different regions. In studies carried out in different countries, Boutoub et al. [46] reported a broader range of diastase activity mean values ranging from 11.93 to 115.89 in honeys of various floral origins in Morocco. In a study by Alves et al. [47], which examined 24 organic honey samples, diastase activity values ranged from 7.6 to 89.6. Similarly, Gomes et al. [17], in a study involving 73 organic honey samples from Portugal, reported diastase activity values between 13.90 and 16.40. In a study conducted in Colombia, Castro et al. [25] analyzed 43 organic and 24 conventional honey samples. The diastase activity of organic honey samples ranged from 6 to 39, whereas that of conventional honey samples ranged from 5 to 25. Do Nascimento et al. [36] reported diastase activity ranging from 7.15 to 57.69 in honey samples. These differences may be attributed to variations in the botanical and geographical origins of the honey samples, as well as differences in production and storage conditions.

#### 2.3.3. HMF Values

HMF is an important parameter indicating the freshness of honey, with low levels indicating its quality. While it forms naturally over time, its formation accelerates when exposed to heat treatment. It is an important criterion widely used in evaluating honey quality [48]. According to the TFC, the HMF content in honey should be a maximum of 40 mg/kg [39]. In the present study, the mean HMF values of organic and conventional honey samples were found to vary between 3.80 and 13.11 mg/kg and 3.24–23.30 mg/kg, respectively (Table 2, Figure 1). The mean HMF values of A_1_ and B_1_ were found to be significantly higher (*p* < 0.05) compared to the A_2_ and B_2_ samples. In contrast, the mean HMF values of C_2_ and D_2_ samples were found to be significantly higher (*p* < 0.05) compared to the C_1_ and D_1_ samples. At the same time, the HMF results of the honey samples were well below the limit value (40 mg/kg) specified in the TFC. These results show that the analyzed honey samples were preserved under appropriate production and storage conditions.

In Türkiye, Gürbüz et al. [49] reported a mean HMF value of 18.50 ± 31.43 mg/kg in 68 honey samples collected from various regions of Türkiye. Çelemli et al. [50] determined that the HMF content of twenty honey samples, including both monofloral and multifloral types produced in Ayder, Rize, ranged from 0.70 to 11.31 mg/kg, with a mean value of 3.80 ± 2.6 mg/kg. Ucuncu et al. [33] reported HMF levels ranging from 0.29 to 19.95 mg/kg, with a mean value of 4.51 ± 4.48 mg/kg, in fifteen flower honey samples obtained from Gümüşhane province. Bideci and Karasalihoğlu [40] reported that HMF levels in honey samples in Kastamonu province ranged from 7.00 to 37.40 mg/kg, with a mean value of 18.61 ± 8.14 mg/kg. In an international study, Castro et al. [25] reported that the HMF content of the investigated organic and conventional honey samples in Colombia ranged from 0 to 46.38 mg/kg and 0 to 75.08 mg/kg, respectively. Moise et al. [35] reported a mean HMF level of 10.00 ± 3.02 mg/kg in heather honey samples originating from Romania. Do Nascimento et al. [36] reported HMF levels ranging from 0.47 to 22.72 mg/kg in honey samples. Gomes et al. [17], in a study involving 73 organic honey samples from Portugal, reported HMF levels ranging from 0.77 to 1.50 mg/kg, with a mean value of 1.14 ± 0.20 mg/kg. Bouddine et al. [24] analyzed organic honey samples and found that HMF values were below the recommended limit of 40 mg/kg.

Diastase activity, together with HMF content, is considered an important indicator of honey freshness [29]. Changes in the characteristics and quality of honey may result from exposure to temperature, humidity, air, and light. Sugar degradation, HMF formation, reduced diastase activity, increased acidity, decreased pH, degradation of phenolic compounds, and color changes are among the alterations that may occur during storage [51]. In the present study, the HMF values were generally observed to be consistent with the diastase numbers of the honey samples.

#### 2.3.4. pH Values

**Figure 2:**
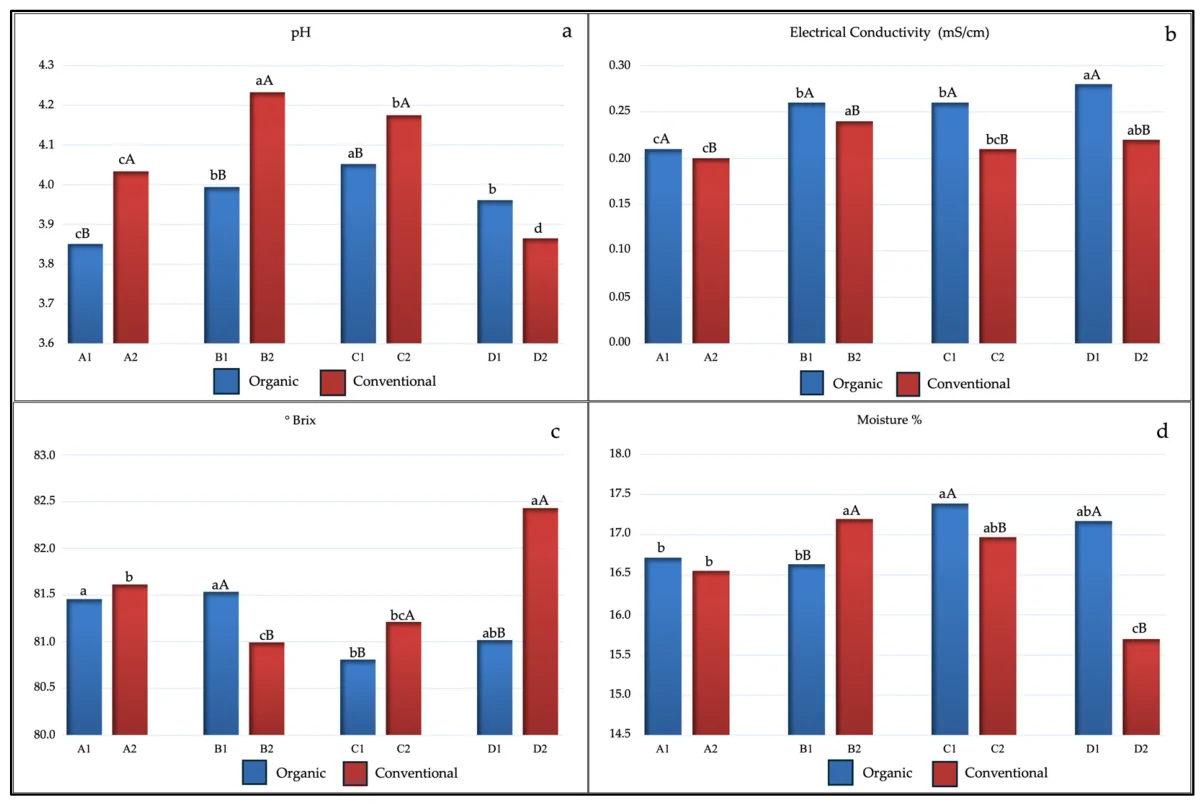
pH values (**a**), electrical conductivity (**b**), °Brix (**c**), and moisture values (**d**) of organic and conventional honey samples. Different lowercase letters (a–d) above the bars indicate statistically significant differences among firms within the same production group (organic or conventional) (*p* < 0.05). Different uppercase letters (A and B) indicate statistically significant differences between the organic and conventional samples of the same firm (*p* < 0.05); the absence of an uppercase letter indicates that the difference between organic and conventional samples for that specific firm is not statistically significant (*p* > 0.05).

The pH of honey serves as a critical indicator for potential microbial growth. While the optimal pH range for most microorganisms varies from 7.2 to 7.4, the characteristically low pH of honey (3.2–4.5) acts as a natural barrier that suppresses microbial survival and growth [27]. In our study, mean pH values were found to range from 3.85 to 4.05 in organic honey samples and from 3.87 to 4.23 in conventional honey samples (Table 3, Figure 2). The pH values of honey samples from firms A_2_, B_2_, and C_2_ were found to be significantly higher (*p* < 0.05) compared to honey samples from firms A_1_, B_1_, and C_1_.

Yurt and Çakır [52] analyzed 30 strained flower honey samples from the Iğdır region of Türkiye and found the mean pH value to be 4.33 ± 0.25. Similarly, a study involving 47 honey samples from the Çorum region of Türkiye reported pH values ranging from 3.55 to 4.20 [53]. When compared with findings from studies conducted in Türkiye, the pH values obtained in the present study are generally consistent with those reported in the literature. In a study conducted by Castro et al. [25] in Colombia, the mean pH value of the organic honey samples was 3.66 ± 0.15, whereas the corresponding value for conventional honey samples was 3.64 ± 0.18. In another study, eight certified organic and conventional honey samples of different geographical and botanical origins (linden, acacia, chestnut, and meadow) were analyzed. The results showed that pH values ranged from 3.41 to 4.34 in organic honeys and from 3.23 to 4.95 in conventional honeys [27]. Moise et al. [35] reported a mean pH value of 4.27 ± 0.07 in heather honey samples originating from Romania. Do Nascimento et al. [36] reported pH levels ranging from 3.58 to 4.95 in honey samples. Gomes et al. [17], in a study involving 73 organic honey samples from Portugal, reported pH levels ranging from 3.45 to 4.00, with a mean value of 3.74 ± 0.13. Overall, the available literature indicates that the pH values of organic and conventional flower honeys are highly consistent with the pH values obtained in the present study.

#### 2.3.5. Electrical Conductivity

As a key parameter of botanical origin and mineral profile, electrical conductivity of honey directly depends on its ionizable components. These include inorganic salts (K, Ca, Mg), organic acids, proteins, and complex sugars that drive charge transport within the aqueous matrix [54]. According to the TFC, electrical conductivity is specified as at least 0.8 mS/cm for honeydew honey and at most 0.8 mS/cm for floral honey [39]. In our study, it was determined that mean electrical conductivity values ranged from 0.21 to 0.28 mS/cm in organic honey samples and from 0.20 to 0.24 mS/cm in conventional honey samples (Table 3, Figure 2). All honey samples analyzed in the present study were found to comply with the limits specified in the TFC. All organic honey samples were found to have significantly higher electrical conductivity values (*p* < 0.05) compared to conventional honey samples of the same firms.

In Türkiye, Ucuncu et al. [33] reported electrical conductivity levels ranging from 0.12 to 1.01 mS/cm, with a mean value of 0.35 ± 0.25 mS/cm, in fifteen flower honey samples obtained from Gümüşhane province. Bideci and Karasalihoğlu [40] reported that electrical conductivity in honey samples in Kastamonu province ranged from 0.06 to 2.52 mS/cm, with a mean value of 0.54 ± 0.38 mS/cm. Yurt and Çakır [52] analyzed 30 strained flower honey samples from the Iğdır region and found the mean electrical conductivity value to be 0.17 ± 0.15 mS/cm. Similarly, flower honeys collected from the Bayburt region were reported to exhibit electrical conductivity values between 0.28 and 0.71 mS/cm [55]. Overall, the electrical conductivity values obtained in the present study were generally lower than those reported in the literature. These relatively low electrical conductivity values may be attributed to the predominance of different floral sources in the analyzed honey samples. In studies conducted in other countries, Bouddine et al. [24] analyzed 23 organic honey samples derived from various floral sources and collected from hives located in different ecogeographical regions of northern, central, and southern Morocco. The electrical conductivity values of the honey samples ranged from 100 to 820 µS/cm. Gomes et al. [17] analyzed 73 organic honey samples in Portugal and reported electrical conductivity values ranging from 0.09 to 0.43 mS/cm. Moise et al. [35] reported a mean electrical conductivity value of 0.66 ± 0.07 mS/cm in heather honey samples originating from Romania.

#### 2.3.6. °Brix Values

Soluble solids content is expressed in Brix degree (°Brix or %Brix) [56]. The mean °Brix values were found to range from 80.81 to 81.54% in organic honey samples and 80.99–82.43% in conventional honey samples (Table 3, Figure 2). Among the analyzed honey samples, the highest mean °Brix value was detected in honey samples from firm D_2_, while the lowest value was observed in honey samples from firm C_1_. The °Brix values of honey samples from firms C_2_ and D_2_ were significantly higher (*p* < 0.05) than those of honey samples from firms C_1_ and D_1_.

In Türkiye, Yurt and Çakır [52] analyzed 30 strained flower honey samples from the Iğdır region and found the mean °Brix value to be 83.30 ± 0.97%. Güzel and Bahçeci [53] analyzed 47 honey samples from the Çorum region and reported °Brix values ranging from 76.8 to 83.8, with a mean of 81.5. In a study conducted by Stanojević et al. [27], eight honey samples obtained from certified beekeepers in the Balkans, including four organic and four conventional samples, were analyzed. The honey samples differed in both botanical origin (linden, acacia, chestnut, and meadow) and geographical origin. The authors reported °Brix values ranging from 77.50 to 84.25% in organic honey samples and from 77.16 to 81.00% in conventional honey samples.

#### 2.3.7. Moisture Values

By creating favorable conditions for microbial activity, high moisture content can adversely affect honey quality during storage [57]. According to the TFC, the moisture content of floral honeys, except for heather (*Calluna vulgaris* and *Erica* spp.), should not exceed 20% [39]. In our study, the moisture values of all honey samples remained below the maximum value specified in the TFC. It was determined that the mean moisture values in organic honey samples ranged from 16.63 to 17.39%, while in conventional honey samples the mean moisture values ranged from 15.70 to 17.20% (Table 3, Figure 2). The highest mean moisture value was found in organic honey from firm C_1_, and the lowest mean moisture value was found in conventional honey from firm D_2_. Furthermore, it was found that honey samples from firms C_1_ and D_1_ had significantly higher values (*p* < 0.05) compared to honey samples from firms C_2_ and D_2_. The moisture content of honey can be influenced by various factors, including environmental conditions, harvesting period, the degree of maturity achieved in the hive, the natural moisture content of the nectar, and practices applied during the extraction and storage of honey [24]. It is stated that organic honey has a lower viscosity due to less internal friction between molecules, which can lead to a higher moisture content [58].

In studies conducted in Türkiye, Ucuncu et al. [33] reported moisture levels ranging from 15.20 to 20.50 g/100 g, with a mean value of 17.44 ± 1.51 g/100 g, in fifteen flower honey samples obtained from Gümüşhane province. Akgün et al. [29] reported moisture levels ranging from 14.45 to 21.62%, with a mean value of 18.43 ± 1.82% in honey samples obtained from Ordu province. Bideci and Karasalihoğlu [40] reported that moisture level in honey samples in Kastamonu province ranged from 1.84 to 20.00%, with a mean value of 17.32 ± 3.91%. Yurt and Çakır [52] analyzed 30 strained flower honey samples from the Iğdır region and found the mean moisture content to be 15.06 ± 0.75%. Güzel and Bahçeci [53] analyzed 47 honey samples from the Çorum region and reported moisture values ranging from 14.5% to 21.7%, with a mean of 16.9%.

In international studies, Gomes et al. [17], in a study involving 73 organic honey samples from Portugal, reported a mean moisture content of 17.17 ± 0.62%. Stanojević et al. [27] observed variations in moisture content between organic and conventional honey samples of the same botanical origin. The authors reported that the moisture content of the analyzed samples varied between 14.43% and 18.39%. Similarly, Bouddine et al. [24] reported moisture values ranging from 14.2% to 19.4% in 23 organic honey samples collected in Morocco. The moisture values obtained in the present study are consistent with those reported in the literature and further demonstrate that honey moisture content can vary over a broad range.

#### 2.3.8. Color

Honey color, a prominent physical characteristic, and sensory attributes fundamentally determine consumer acceptance [27]. Honey color is generally classified according to the Pfund and Lovibond scales [59].

Based on the Pfund scale analysis of organic honey, the majority of samples from firms A_1_ and C_1_ fell into the ‘white’ color group, whereas firm B_1_ displayed a predominance of ‘extra light amber ’, and all firm D_1_ samples were classified as ‘light amber’. Evaluation of conventional honey samples based on the Pfund scale revealed that all samples from firms A_2_ and C_2_ fell into the ‘white’ color group, demonstrating a highly notable color homogeneity. In honey firm B_1_ and D_2_, both “extra light amber” and “light amber” color tones were found (Table 4).

In studies investigating honey color, Halagarda et al. [34] evaluated 22 honey types obtained directly from beehives and organic food markets and reported distinct differences in color parameters among honey samples, suggesting that these differences were primarily associated with botanical origin. Boutoub et al. [46] determined that the mean color of the seven analyzed honey samples ranged between amber and dark amber, with the former observed in only one sample and the latter in the remaining six samples. Previous studies have consistently shown that darker-colored honey samples generally contain higher levels of phenolic compounds than lighter-colored honeys [27,60,61]. When the color distribution and total phenolic content results obtained in the present study are evaluated together, a clear relationship between honey color and phenolic compound content can be observed. According to the color distribution data, honey samples from producers B and D, particularly those classified as extra light amber and light amber, generally contained higher amounts of total phenolic compounds. In contrast, lighter-colored honey samples (white and extra light amber) were predominantly observed in producers A and C and were characterized by lower phenolic contents. These findings are consistent with the total phenolic content results. Indeed, samples from producers B and D, especially within the conventional group, exhibited higher total phenolic contents and were more frequently classified within the darker color categories. Such color variations can be attributed to diverse color pigments derived from phenolic compounds and minerals in honey, which fluctuate depending on its botanical and geographical origins [43]. The relationship observed in the present study between the color distribution of organic and conventional honey samples and their total phenolic content is consistent with findings reported in the literature.

#### 2.3.9. Principal Component Analysis (PCA), Hierarchical Clustering Analysis (HCA) and Correlation Analysis

The unique characteristics of each honey type may stem from the presence or absence of specific physicochemical parameters. In such cases, other parameters can also be affected, ultimately altering the overall quality of the honey [62]. PCA is a statistical tool widely used to compress datasets and evaluate the most relevant information [63]. The Kaiser criterion, which selects Principal Components (PCs) with eigenvalues greater than 1 [64], was used in selecting the number of PCs (Table 5).

**Figure 3:**
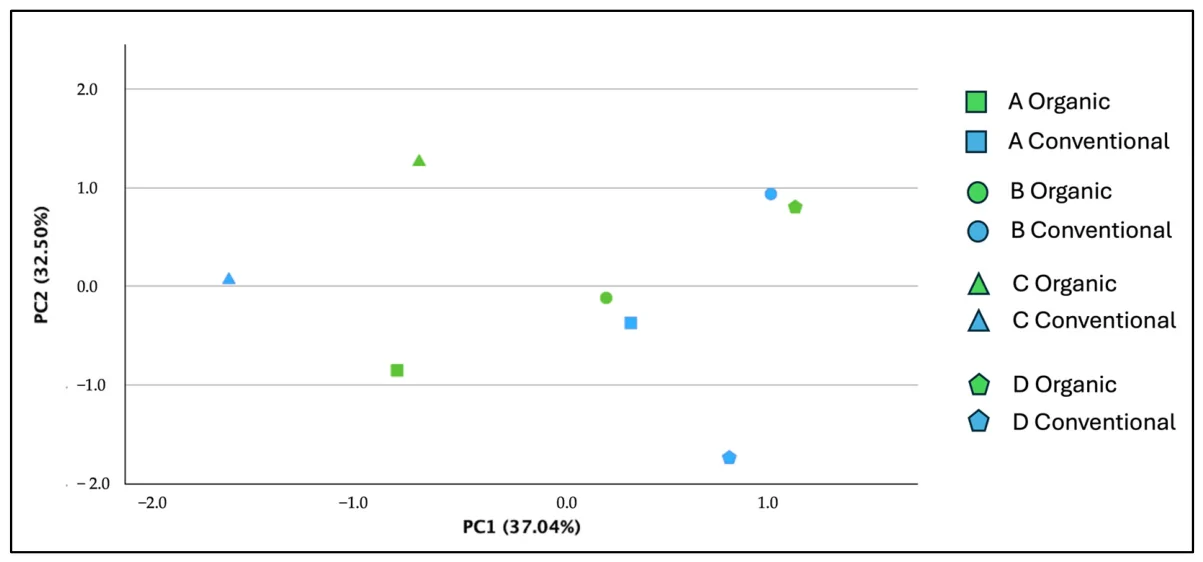
PCA score plot of the honey samples based on the first two principal components.⁠

According to the PCA (Figure 3), conventional honey of B firm and organic honey of D firm are chemically very close in profile, exhibiting high total phenolic content and low HMF values. When examining the organic and conventional honeys of C firm on the graph, they were found to have the highest HMF values and the lowest antioxidant and phenolic contents compared to all other honeys. Conventional honey of D firm is rich in bioactivity; however, it is the honey with the highest °Brix value and the lowest moisture content. In contrast, organic honey of D firm showed an increased moisture content and a decreased °Brix value, while its antioxidant quality remained the same. Conventional honey of A firm clustered right next to organic honey of B firm and could be described as being of average quality. Organic honey of A firm, on the other hand, is observed to have a higher HMF and lower total phenolic content compared to its conventional counterpart. As a result of the PCA, it can be concluded that firm-based production is dominant in terms of the quality and composition of honeys rather than organic or conventional production methods. For instance, while organic production improves the quality and moisture balance in D firm, the conventional product of B firm is already at a level close to the highest quality organic honeys. C firm, regardless of the organic or conventional production method, completely diverges from the other firms with its high HMF and low bioactive profile.

**Figure 4:**
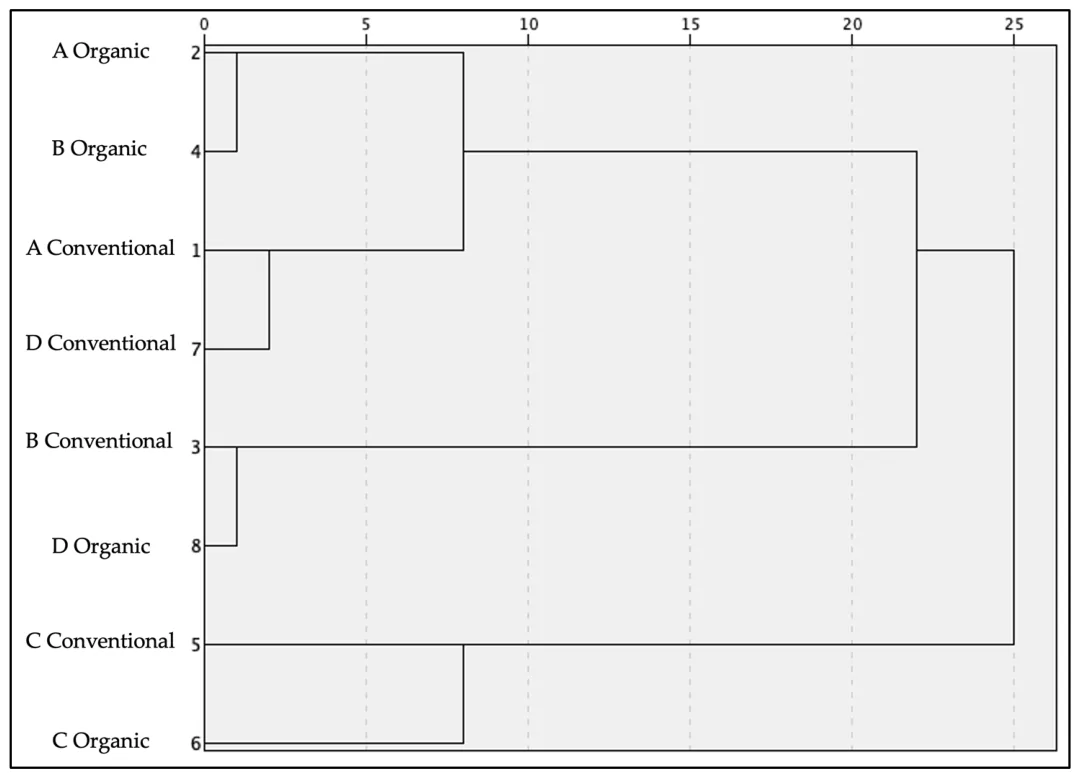
Dendrogram obtained from HCA of honey samples based on their physicochemical and bioactive properties. A, B, C, and D represent the commercial honey firms.

When the hierarchical cluster graph (Figure 4) is evaluated, it is seen that the dataset is divided into 3 main clusters. Cluster 1: Organic honey from A and B firms and conventional honey from A and D firms, which are similar in profile. Cluster 2: Conventional honey from B firm and organic honey from D firm are similar in chemical profile. Cluster 3: Conventional and organic honey from C firm have completely different chemical characteristics from the honeys of the other firms.

When the correlation analysis (Table 6) is evaluated, across all honey samples, HMF exhibited negative correlations with five parameters: electrical conductivity, proline, total phenolic content, TAS, and TOS. In a study conducted by Kıvrak et al. [65], a negative correlation was reported between the HMF content of honey and both its total phenolic content and proline levels. The authors noted that a decrease in total phenolic content and proline, accompanying higher HMF levels, serves as an indicator of reduced honey quality, often driven by thermal treatment, which accelerates the acid-catalyzed dehydration of hexoses. These findings are consistent with the significant negative correlations observed between HMF and both total phenolic content and proline in our current study.

In addition, °Brix values showed negative correlations with both pH and moisture content. Also, there was a high negative correlation between moisture content and °Brix. Similarly to our study, Nisha and Tarsikka [66] reported that increasing moisture content in honey decreases its refractive index and °Brix. A similar correlation was also reported by Anupama et al. [67] and Kobacy et al. [68]. Nidhi et al. [69] found a negative correlation between °Brix level and pH, attributing this to a decrease in pH as total sugar content increases. The °Brix level is related to the moisture content of honey; as the moisture content increases, the °Brix value decreases. A high °Brix value is an important criterion for the quality of honey, especially during the market or storage period [70]. Rivera-Mondragón et al. [71] highlighted that the negative correlation observed between moisture and °Brix indicates an inverse relationship with water content. They noted that this may result from harvesting under elevated humidity conditions or could be associated with the botanical source of the honey.

At the same time, it is observed that as HMF, an undesirable compound in honey, increases in both organic and conventional honeys, the TAS of the honey decreases significantly. The antioxidant activity of honey is also affected by Maillard reaction products [72].

As the proline level increases in organic honeys, diastase enzyme activity also increases in parallel (r = 0.390 **, *p* = 0.007). Proline and diastase are bee-derived components, and the correlation between them has been reported as moderately positive in the study by Tkáč et al. [73], similar to our results.

A negative relationship is found between diastase and HMF in conventional honeys (r = −0.675 **, *p* < 0.001). Two physicochemical parameters of honey affected by heat treatment are HMF content and diastase number. As a result of heat treatment, the diastase number of honey tends to decrease, while the HMF content tends to increase, and therefore there is a negative correlation between them [62]. Pasias et al. [74] also stated that heat treatment has negative effects on diastase activity. Similarly to our study, Partigniani et al. [75] reported a significant negative correlation (*p* ≤ 0.05) between HMF content and diastase activity in their study. Chaikham et al. [76] reported a correlation between diastase activity and HMF, stating that heat treatment increased HMF and decreased diastase activity.

A positive relationship is also observed between total phenolic content and proline in honey samples (r = 0.702 ** in conventional, r = 0.538 ** in organic). Da Costa and Toro [77] reported that proline has a strong correlation with phenolic compounds, which may contribute to the antioxidant activity of the amino acid content in honey.

Significant positive correlations between TOS and TAS, as well as between TOS and total phenolic content, were observed across all honey samples. These findings are consistent with the results reported by Herken et al. [20], who also identified significant positive correlations between TOS and TAS, and between TOS and total phenolic content. The authors reported that the positive correlation between antioxidant and oxidant parameters indicates the presence of intrinsic mechanisms in honey that regulate the balance of ROS.

Total phenolic content was positively correlated with electrical conductivity in all honey samples. This finding is consistent with the results reported by Albu et al. [78], Tkáč et al. [73], and Tananaki et al. [79], who also observed a positive correlation between total phenolic content and electrical conductivity in honey.

Similarly, a statistically significant positive correlation was observed between total phenolic content and proline, which is consistent with the findings of Islam et al. [80], Da Costa and Toro [77], and Tkáč et al. [73].

Furthermore, pH exhibited positive correlations with both electrical conductivity and moisture content. Consistent with the present findings, a correlation between electrical conductivity and pH in honey was reported by Thakur et al. [81]. The electrical conductivity of honey is determined by the content of inorganic salts, organic acids, proteins, complex sugars, and minerals in the sample and may therefore influence its pH [81,82]. Krishnasree and Ukkuru [62] and Manikavasakam et al. [83] observed a positive correlation between pH and moisture in honey samples. It has been noted that there is a strong relationship between moisture content and pH, and that when the moisture content of honey increases, it triggers the growth of microorganisms, which leads to the fermentation of the honey. Consequently, when honey ferments, its pH and free acidity automatically increase [83].

Spearman correlation analyses revealed that organic and conventional production processes change the internal dynamics of honeys.

## 3. Materials and Methods

### 3.1. Honey Sample Collection

In the present study, ninety-two floral honey samples produced between January and December 2024 and obtained from commercial retail sources in Türkiye were used. According to the product label information, the production regions indicated for the honey samples included Central Anatolia, Eastern Anatolia, and the Mediterranean regions of Türkiye; some samples indicated more than one production region.

The honey samples used in the study were obtained from four different firms (A, B, C, and D) producing organic and conventional honey. A total of 92 honey samples were analyzed in this study, comprising 46 organic and 46 conventional variants obtained across four different firms. Specifically, 24 samples (12 organic and 12 conventional) were collected from each of firms A, B, and C, while 20 samples (10 organic and 10 conventional) were collected from firm D. For tracking purposes, organic samples were coded as A_1_–D_1_, and conventional samples as A_2_–D_2_. Honey samples classified as organic possessed organic farming certification. All samples were stored in their original containers, in the dark, at room temperature until analysis. Moisture and °Brix analyses were performed in triplicate, while all other measurements were carried out in duplicate.

### 3.2. Determination of Total Antioxidant Status (TAS) and Total Oxidant Status (TOS)

TAS and TOS levels of honey samples were determined using commercial assay kits (Rel Assay, Gaziantep, Türkiye) according to established methodologies [20,84,85]. The TAS assay relied on the decolorization of the stable ABTS radical cation by antioxidants, with results expressed as mmol Trolox equivalent/L. Conversely, the TOS assay was based on the oxidant-mediated oxidation of the ferrous ion–o-dianisidine complex to ferric ions, enhanced by glycerol molecules, which subsequently formed a colored complex with xylenol orange under acidic conditions. TOS values were expressed as μmol H_2_O_2_ equivalent/L. For sample preparation, 0.1 g of honey was weighed into a microtube, mixed with 900 µL of 500 mmol phosphate buffer (pH 7.40), and vortexed for 30 s until complete homogenization. The mixture was then centrifuged at 10,000 rpm for 5 min at +4 °C, and the resulting supernatant was collected for immediate spectrophotometric analysis.

### 3.3. Determination of Total Phenolic Content

Total phenolic content of honey samples was evaluated using the Folin–Ciocalteu method [20,86,87]. For this purpose, 0.1 g of honey sample was weighed and transferred to a microcentrifuge tube, and 900 µL of 500 mM, pH 7.40 phosphate buffer was added. The sample was vortexed for 30 s until completely homogenized, and then centrifuged at 10,000 rpm for 5 min at +4 °C. Fifty microliters (50 µL) of the liquid phase (supernatant) formed at the top of the tube after centrifugation was taken, and 200 µL of Folin–Ciocalteu reagent was added. After vortexing the sample, 400 µL of 10% sodium carbonate (Na_2_CO_3_) solution was added and vortexed again. The reaction mixture was incubated in the dark at room temperature for 30 min, and then absorbance values were measured using a spectrophotometer at a wavelength of 760 nm. The total phenolic content was calculated using the gallic acid standard curve, and the results were expressed in mg GAE/kg honey.

### 3.4. Proline Analysis

Proline levels in honey samples were measured using commercial kits (Otto Scientific, Ankara, Türkiye). The method is based on spectrophotometric methods recommended by the International Bee Products Commission [88]. Honey samples were processed and analyzed following the guidelines recommended by the kit manufacturer. The absorbances were read at 514 nm in the spectrophotometer, and calculations were performed (mg proline/kg honey).

### 3.5. Measurement of Diastase Activity

Diastase activity is determined by testing the enzymatic effect of honey in a solution. Since diastase contains an enzyme that breaks down starch, the time it takes for starch to break down is measured. In this study, diastase activity determination was performed using commercial kits, in accordance with the harmonized analysis methods of the International Honey Commission and the TSI Honey-diastase activity determination [88,89]. Honey samples were prepared according to the kit guidelines, and the spectrophotometer measurement of the samples was performed at 660 nm.

### 3.6. Hydroxymethylfurfural (HMF) Analysis

HMF analysis in honey was performed according to the spectrophotometric method described by Official Methods of Analysis of AOAC International [90]. Briefly, 5 g of honey sample was weighed into a 50 mL tube, and 25 mL of distilled water was added. Then, 0.5 mL of Carrez I and 0.5 mL of Carrez II solutions were added to the honey solution to precipitate the proteins. The solution was diluted to 50 mL with distilled water and homogenized by vortexing for 20 s. The solution was filtered through coarse filter paper, and the first 10 mL was discarded. Afterwards, 5 mL of filtrate was collected into two test tubes, followed by the addition of 5 mL of sodium bisulfite solution to the reference tube and 5 mL of distilled water to the other tube, and both were vortexed. Absorbances were measured at 284 nm and at 336 nm in a spectrophotometer. The results were calculated as mg/kg.

### 3.7. pH and Electrical Conductivity Measurement

pH and electrical conductivity measurements of honey samples were performed using a Hanna HI 98130 digital pH/EC/TDS meter (Cluj, Romania). pH and electrical conductivity measurements of honey samples were performed based on the standards of Turkish Standards Institute [91,92]. Measurements were taken at room temperature, in duplicate for each sample [93,94]. The electrical conductivity of each honey sample was expressed as mS/cm.

### 3.8. Color Measurement

Color analyses of honey samples were determined according to the spectrophotometric method described by White [95] and Paula et al. [96]. Briefly, 5 g of honey sample was weighed and dissolved in 10 mL of distilled water. The absorbance was read at 635 nm in a spectrophotometer. The color of the honey samples on the mm Pfund scale was calculated based on the converted absorbance values.

### 3.9. °Brix and Moisture Measurement

Water-soluble dry matter (%Brix or °Brix) analysis of honey samples was performed using a digital refractometer (Milwaukee MA871 Digital Refractometer, North Carolina, NC, USA). Analyses were measured as % dry matter content at 20 °C. Honey samples were dropped onto the prism section of the refractometer, and the °Brix value was read directly from the device’s screen. Three replicates were taken from each sample, and the results were reported as averages [97]. Moisture content of honey samples was calculated using a refractometric index [98].

### 3.10. Statistical Analysis

Statistical analyses of the data obtained from honey sample analyses were performed using SPSS software (IBM SPSS Statistics Version 31). The normality of the data distribution was assessed using the Shapiro–Wilk test. For parameters showing normal distribution, differences between groups were evaluated using the independent samples *t*-test and one-way analysis of variance (one-way ANOVA). Following one-way ANOVA, Tukey’s post hoc test was applied to determine the source of significant differences among groups. For data that did not show normal distribution, non-parametric tests including the Mann–Whitney U test and Kruskal–Wallis test were used to evaluate statistical differences between groups [99]. Principal component analysis (PCA) and hierarchical clustering analysis (HCA) were applied to the chemical and physicochemical data of organic and conventional honeys. Spearman rank correlation analysis was used to determine the correlation coefficients between the variables.

## 4. Conclusions

In the present study, TAS, TOS, total phenolic content, proline, diastase activity, and certain physicochemical properties of organic and conventional floral honeys marketed in Türkiye were evaluated. The mean results demonstrated that the analyzed honey samples generally complied with the quality criteria specified in the TFC. When the results were evaluated, the diastase activity, proline content, and HMF levels determined in the honey samples indicated that the products were generally produced and stored under appropriate conditions. In addition, the electrical conductivity, pH, moisture, and °Brix values were found to be largely consistent with the ranges reported in the literature. Evaluation of the antioxidant parameters revealed that the honey samples possessed considerable antioxidant capacity, which was associated with their total phenolic content. Furthermore, a relationship was observed between honey color and total phenolic content, with darker-colored samples generally exhibiting higher phenolic concentrations. The results demonstrate that the floral honey samples investigated in this study generally comply with essential quality standards and exhibit antioxidant potential. Analytical controls of honey, as a widely consumed functional and natural food, play a critical role in safeguarding food quality and public health. Future studies involving larger sample sizes and detailed characterization of individual phenolic compounds would contribute to a more comprehensive understanding of the functional properties of honey.

## Figures and Tables

**Table 1:** TAS, TOS, and total phenolic contents of honey samples (Mean ± SE).

Honey Samples	Firms	N	TAS (mmol Trolox Equivalent/L)	TOS (µmol H_2_O_2_ Equivalent/L)	Total Phenolic Content (mg GAE/kg)
Organic	A_1_	12	0.76 ± 0.02 ^b^	12.03 ± 1.71 ^bB^	101.03 ± 4.68 ^bB^
	B_1_	12	0.93 ± 0.02 ^aA^	33.12 ± 2.23 ^aB^	106.93 ± 4.10 ^abB^
	C_1_	12	0.70 ± 0.03 ^bA^	13.48 ± 3.10 ^bB^	97.17 ± 4.06 ^b^
	D_1_	10	0.81 ± 0.04 ^ab^	56.76 ± 5.33 ^aA^	124.14 ± 4.73 ^aB^
Conventional	A_2_	12	0.80 ± 0.04 ^a^	28.57 ± 2.13 ^aA^	134.51 ± 6.50 ^aA^
	B_2_	12	0.88 ± 0.02 ^aB^	53.43 ± 3.24 ^aA^	153.00 ± 6.51 ^aA^
	C_2_	12	0.48 ± 0.02 ^bB^	14.26 ± 0.62 ^bA^	98.60 ± 5.84 ^b^
	D_2_	10	0.89 ± 0.03 ^a^	33.38 ± 1.77 ^aB^	149.09 ± 8.00 ^aA^

Different lowercase superscript letters (^a, b^) within the same column indicate statistically significant differences among firms within the same production group (organic or conventional) (*p* < 0.05). Different uppercase superscript letters (^A, B^) indicate statistically significant differences between the organic and conventional samples of the same firm (*p* < 0.05); the absence of an uppercase letter indicates that the difference between organic and conventional samples for that specific firm is not statistically significant (*p* > 0.05).

**Table 2:** Proline, diastase, and HMF values of honey samples (Mean ± SE).

Honey Samples	Firms	N	Proline (mg Proline/kg Honey)	Diastase Number	HMF (mg/kg)
Organic	A_1_	12	314.27 ± 16.03 ^cB^	8.57 ± 0.00 ^cB^	12.85 ± 1.68 ^abA^
	B_1_	12	355.89 ± 23.66 ^cB^	15.00 ± 1.51 ^b^	7.46 ± 0.60 ^bcA^
	C_1_	12	479.50 ± 26.05 ^bA^	28.50 ± 1.50 ^aA^	13.11 ± 1.28 ^aB^
	D_1_	10	699.81 ± 47.47 ^a^	12.90 ± 0.46 ^b^	3.80 ± 0.59 ^cB^
Conventional	A_2_	12	531.09 ± 51.76 ^abA^	18.33 ± 1.28 ^aA^	5.54 ± 0.25 ^bcB^
	B_2_	12	538.07 ± 27.26 ^abA^	17.92 ± 0.74 ^a^	3.24 ± 0.23 ^cB^
	C_2_	12	384.05 ± 9.85 ^bB^	12.08 ± 0.97 ^bB^	23.30 ± 0.98 ^aA^
	D_2_	10	589.16 ± 62.72 ^a^	12.00 ± 0.82 ^b^	13.23 ± 0.23 ^abA^

Different lowercase superscript letters (^a–c^) within the same column indicate statistically significant differences among firms within the same production group (organic or conventional) (*p* < 0.05). Different uppercase superscript letters (^A, B^) indicate statistically significant differences between the organic and conventional samples of the same firm (*p* < 0.05); the absence of an uppercase letter indicates that the difference between organic and conventional samples for that specific firm is not statistically significant (*p* > 0.05).

**Table 3:** pH, electrical conductivity, °brix and moisture values of honey samples (Mean ± SE).

Honey Samples	Firms	N	pH	Electrical Conductivity (mS/cm)	°Brix (%)	Moisture (%)
Organic	A_1_	12	3.85 ± 0.01 ^cB^	0.21 ± 0.001 ^cA^	81.46 ± 0.13 ^a^	16.72 ± 0.14 ^b^
	B_1_	12	3.99 ± 0.01 ^bB^	0.26 ± 0.003 ^bA^	81.54 ± 0.13 ^aA^	16.63 ± 0.14 ^bB^
	C_1_	12	4.05 ± 0.02 ^aB^	0.26 ± 0.004 ^bA^	80.81 ± 0.08 ^bB^	17.39 ± 0.08 ^aA^
	D_1_	10	3.96 ± 0.02 ^b^	0.28 ± 0.002 ^aA^	81.02 ± 0.15 ^abB^	17.17 ± 0.16 ^abA^
Conventional	A_2_	12	4.03 ± 0.00 ^cA^	0.20 ± 0.002 ^cB^	81.61 ± 0.11 ^b^	16.55 ± 0.12 ^b^
	B_2_	12	4.23 ± 0.00 ^aA^	0.24 ± 0.005 ^aB^	80.99 ± 0.09 ^cB^	17.20 ± 0.10 ^aA^
	C_2_	12	4.18 ± 0.01 ^bA^	0.21 ± 0.003 ^bcB^	81.21 ± 0.08 ^bcA^	16.97 ± 0.09 ^abB^
	D_2_	10	3.87 ± 0.00 ^d^	0.22 ± 0.001 ^abB^	82.43 ± 0.11 ^aA^	15.70 ± 0.11 ^cB^

Different lowercase superscript letters (^a–d^) within the same column indicate statistically significant differences among firms within the same production group (organic or conventional) (*p* < 0.05). Different uppercase superscript letters (^A, B^) indicate statistically significant differences between the organic and conventional samples of the same firm (*p* < 0.05); the absence of an uppercase letter indicates that the difference between organic and conventional samples for that specific firm is not statistically significant (*p* > 0.05).

**Table 4:** Color classification of honey samples.

Honey Samples	Firms	N	Pfund Color Grade						
			Water White	Extra White	White	Extra Light Amber	Light Amber	Amber	Dark Amber
Organic	A_1_	12			11	1			
	B_1_	12				11	1		
	C_1_	12			9	3			
	D_1_	10					10		
Conventional	A_2_	12			12				
	B_2_	12			2	10			
	C_2_	12			12				
	D_2_	10				6	4		

**Table 5:** PCA of physicochemical and bioactive properties of honey samples: eigenvectors, eigenvalues, and explained variance.

Kaiser Criterion			
	Component		
	1	2	3
TAS	0.829	−0.202	−0.327
TOS	0.895	0.255	0.134
Total_Phenolic	0.815	−0.213	0.507
Proline	0.748	0.188	0.214
Diastase	−0.012	0.639	0.002
HMF	−0.848	−0.268	0.164
pH	−0.133	0.639	0.672
Electrical_Conductivity	0.398	0.625	−0.536
°Brix	0.217	−0.946	0.042
Moisture	−0.219	0.944	−0.047
Eigenvalues Total	3.704	3.250	1.197
% of Variance	37.036	32.501	11.971
Cumulative %	37.036	69.536	81.508

**Table 6:** Spearman correlation matrix between physicochemical and bioactive parameters of organic and conventional honeys.

		Electrical Conductivity	Diastase	HMF	Proline	Total Phenolic Content	°Brix	Moisture	pH	TAS	TOS
Electrical Conductivity	*r*		0.002	−0.298 *	0.217	0.363 *	−0.083	0.083	0.347 *	0.390 **	0.520 **
	*p*		0.992	0.044	0.147	0.013	0.585	0.585	0.018	0.007	<0.001
Diastase	*r*	0.549 **		−0.675 **	0.128	0.354 *	−0.349 *	0.349 *	0.318 *	0.288	0.448 **
	*p*	<0.001		<0.001	0.397	0.016	0.017	0.017	0.031	0.053	0.002
HMF	*r*	−0.524 **	−0.002		−0.430 **	−0.549 **	0.254	−0.254	−0.359 *	−0.548 **	−0.716 **
	*p*	<0.001	0.989		0.003	<0.001	0.089	0.089	0.014	<0.001	<0.001
Proline	*r*	0.676 **	0.390 **	−0.383 **		0.702 **	0.056	−0.056	−0.142	0.515 **	0.495 **
	*p*	<0.001	0.007	0.009		<0.001	0.713	0.713	0.346	<0.001	<0.001
Total Phenolic Content	*r*	0.353 *	−0.164	−0.435 **	0.538 **		0.060	−0.060	0.006	0.541 **	0.632 **
	*p*	0.016	0.275	0.003	<0.001		0.690	0.690	0.966	<0.001	<0.001
°Brix	*r*	−0.209	−0.213	−0.068	−0.090	0.156		−1.000 **	−0.583 **	0.115	−0.027
	*p*	0.163	0.155	0.654	0.552	0.301			<0.001	0.448	0.859
Moisture	*r*	0.219	0.223	0.063	0.100	−0.152	−0.997 **		0.583 **	−0.115	0.027
	*p*	0.144	0.136	0.679	0.508	0.315	<0.001		<0.001	0.448	0.859
pH	*r*	0.390 **	0.670 **	−0.028	0.257	−0.118	−0.389 **	0.394 **		−0.155	0.131
	*p*	0.007	<0.001	0.851	0.085	0.433	0.008	0.007		0.303	0.386
TAS	*r*	0.029	−0.093	−0.376 **	−0.291 *	0.139	0.267	−0.275	−0.051		0.697 **
	*p*	0.847	0.540	0.010	0.049	0.358	0.073	0.064	0.737		<0.001
TOS	*r*	0.474 **	0.072	−0.662 **	0.277	0.437 **	0.109	−0.119	0.080	0.582 **	
	*p*	<0.001	0.636	<0.001	0.063	0.002	0.469	0.430	0.599	<0.001	

Values below the diagonal represent organic honeys, while values above the diagonal represent conventional honeys. r: Spearman correlation coefficient; *p*: Significance value. *⁠ Correlation is significant at the 0.05 level (2-tailed). **⁠ Correlation is significant at the 0.01 level (2-tailed). Green shading indicates positive correlations, whereas red shading indicates negative correlations, with color intensity reflecting the strength of the correlation.

## Data Availability

The original contributions presented in the study are included in the article; further inquiries can be directed to the corresponding author.
